# Three-Dimensional Bioprinting of Ovine Aortic Valve Endothelial and Interstitial Cells for the Development of Multicellular Tissue Engineered Tissue Constructs

**DOI:** 10.3390/bioengineering10070787

**Published:** 2023-06-30

**Authors:** Moritz Benjamin Immohr, Helena Lauren Teichert, Fabió dos Santos Adrego, Vera Schmidt, Yukiharu Sugimura, Sebastian Johannes Bauer, Mareike Barth, Artur Lichtenberg, Payam Akhyari

**Affiliations:** 1 Department of Cardiac Surgery, Medical Faculty and University Hospital Düsseldorf, Heinrich-Heine-University Düsseldorf, 40225 Duesseldorf, Germany; mimmohr@ukaachen.de (M.B.I.);; 2 Department of Cardiac Surgery, Medical Faculty, RWTH Aachen University, 52074 Aachen, Germany

**Keywords:** calcific aortic valve disease, valvular interstitial cells, valvular endothelial cells, tissue engineering, regenerative medicine, 3D printing, bioprinting, 3D cell culture

## Abstract

To investigate the pathogenic mechanisms of calcified aortic valve disease (CAVD), it is necessary to develop a new three-dimensional model that contains valvular interstitial cells (VIC) and valvular endothelial cells (VEC). For this purpose, ovine aortic valves were processed to isolate VIC and VEC that were dissolved in an alginate/gelatin hydrogel. A 3D-bioprinter (3D-Bioplotter^®^ Developer Series, EnvisionTec, Gladbeck, Germany) was used to print cell-laden tissue constructs containing VIC and VEC which were cultured for up to 21 days. The 3D-architecture, the composition of the culture medium, and the hydrogels were modified, and cell viability was assessed. The composition of the culture medium directly affected the cell viability of the multicellular tissue constructs. Co-culture of VIC and VEC with a mixture of 70% valvular interstitial cell and 30% valvular endothelial cell medium components reached the cell viability best tested with about 60% more living cells compared to pure valvular interstitial cell medium (*p* = 0.02). The tissue constructs retained comparable cell viability after 21 days (*p* = 0.90) with different 3D-architectures, including a “sandwich” and a “tube” design. Good long-term cell viability was confirmed even for thick multilayer multicellular tissue constructs. The 3D-bioprinting of multicellular tissue constructs with VEC and VIC is a successful new technique to design tissue constructs that mimic the structure of the native aortic valve for research applications of aortic valve pathologies.

## 1. Introduction

The complex 3D-architecture of the aortic valve consisting of valvular endothelial cells (VEC), valvular interstitial cells (VIC), and the extracellular matrix (ECM) contributes to the development of calcific aortic valve disease (CAVD) [1,2,3]. Therefore, the pathogenesis of CAVD involving the interplay of the valvular cells and ECM as well as several endogenic and exogenic factors is still not fully understood [1,2].

Consequently, 3D in vitro models that try to mimic the architecture of the native aortic valve and surpass conventional 2D-models have gained increasing interest in cardiovascular research [4,5,6,7,8]. Hereby, 3D-bioprinting offers diverse new possibilities as it can manufacture standardized cell-laden tissue constructs to examine the mechanisms of CAVD [9,10,11,12]. In contrast to conventional 3D-printing that utilizes thermoplastics and is used in cardiovascular surgery for surgical training and operation planning, 3D-bioprinting processes biological inks, namely hydrogels, that can contain cells and components of the extracellular matrix [13,14]. However, shear stress, as well as temperature and pressure fluctuations during the printing process, still limit cell viability, especially for vulnerable cell lines such as valvular endothelial cells [15,16,17]. Therefore, 3D-bioprinted models of the aortic valve have focused only on VIC but have not yet handled VEC [9,10,11,12]. However, outside of aortic valve and CAVD research, successful 3D-bioprinting of stem-cell-derived endothelial cells and human umbilical vein endothelial cells (HUVEC) has been reported [18,19,20]. In addition, multiple other tissue engineering techniques have been established in combination with 3D-bioprinting in all different fields of medicine [21,22,23,24,25].

As the native aortic valve consists of VEC and VIC, combining both cell lines in one co-culture is an essential approach to further develop CAVD research [26,27,28,29,30,31,32,33]. Here, VIC and VEC were shown to communicate with each other and thus affect both the calcification and the endothelial-to-mesenchymal transition of VEC [26,27,28,29,30,31,32,33]. However, none of these co-culture models have used 3D-bioprinting yet and consequently needed multiple complex steps to produce the multicellular tissue construct. Additionally, the culture of VIC and VEC in a combined tissue construct remains challenging, as both cell populations in general have different nutritional demands and require different cell culture mediums for long-term viability.

Therefore, our objective was to establish a novel technique for a combined tissue construct of VEC and VIC co-culture with long-term cell viability by using 3D-bioprinting to mimic the 3D-architecture of the native aortic valve for future applications for CAVD research. Our focus was particularly the technical aspect of the 3D-bioprinted cellular constructs and the achievement of good long-term cell viability in multicellular tissue constructs that could satisfy the different demands of two different cell types in a combined construct. Therefore, we first established the 3D-bioprinting of VEC, then identified the ideal incubation parameters for a co-culture of VIC and VEC and finally established the 3D-bioprinting of the combined multicellular tissue constructs.

## 2. Materials and Methods

**Figure 1:**
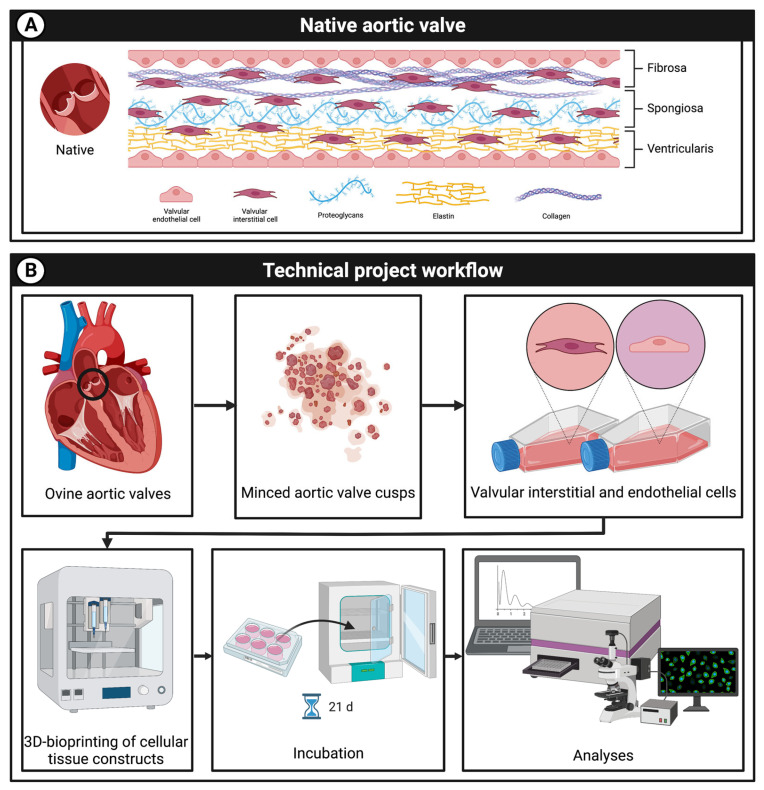
Project overview. (**A**): schematic graphic of the native aortic valve consisting of aortic valve interstitial (VIC) and endothelial cells (VEC) as well as the extracellular matrix with proteoglycans, elastin, and collagen. (**B**): the aim of the research is to develop a multicellular 3D-bioprinted tissue construct containing VIC and VEC mimicking the native aortic valve structure. Therefore, cells were isolated from ovine aortic valves and printed with a 3D-bioprinter; tissue constructs were incubated for up to 21 days and cell viability was analyzed.

In the current project, we aimed to develop a multicellular 3D-bioprinted tissue con-struct mimicking the native aortic valve structure (Figure 1) for future CAVD research ap-plications. Therefore, native adult ovine aortic valve cells were isolated, cultured, 3D-bioprinted in tissue constructs, and evaluated (Figure 1). For the isolation of native adult aortic VEC, ovine hearts of different breeds of the food industry (mainly German Merino landsheep and German blackheaded mutton sheep) were purchased from a slaughterhouse (Laame GmbH & Co. KG, Wuppertal, Germany) and processed immediately after slaughtering. The cusps of the aortic valves were explanted and rinsed in endothelial cell growth medium (MV, C-22020, Promocell GmbH, Heidelberg, Germany). Subsequently, the cusps were incubated for 5 min at 1 mg/mL collagenase A (10103578001, Roche, Basel, Switzerland) in MV at 37 °C. Cell suspension was centrifuged at 1500 rpm for 5 min and then resuspended in MV and incubated at 37 °C and 5% CO_2_ in cell culture flasks. The culture medium was changed three times a week. As soon as cells were confluent, they were washed twice with Dulbecco’s balanced salt solution (DPBS, 14190094, Gibco™, ThermoFisher Scientific, Waltham, MA, USA), incubated with trypsin (252000565, Gibco™, ThermoFisher Scientific, Waltham, MA, USA) for 3 min at 37 °C and 5% CO_2_, and passaged. For experiments, cells in passages six to ten were used.

A similar protocol was used for the isolation of native adult ovine VIC. The dissected aortic valve cusps were rinsed in DPBS, minced, and incubated in Dulbecco’s modified eagle’s medium (DMEM, D6546, Sigma-Aldrich, St. Louis, MO, USA) containing 10% fetal bovine serum (10270106, Gibco™, ThermoFisher Scientific, Waltham, MA, USA), 1% penicillin/streptomycin (15140122, Gibco™, ThermoFisher Scientific), 1% glutamine (25030025, Gibco™, ThermoFisher Scientific, Waltham, MA, USA), 1% amphotericin B (62044908, Gilead Sciences, Foster City, CA, USA), and 1% non-essential amino acids (M7145, Sigma-Aldrich, St. Louis, MO, USA) at 37 °C and 5% CO_2_ in cell culture flasks. Similar to the VIC, the cell culture medium was changed three times a week and cells of passages six to ten were used for the experiments.

For 3D-bioprinting, alginate (alginic acid sodium salt of brown algae, medium viscosity, A2033, Sigma-Aldrich, St. Louis, MO, USA) and gelatin (gelatin from porcine skin, gel strength 300, Type A, G2500, Sigma-Aldrich, St. Louis, MO, USA) hydrogels were prepared as previously described by our group [12]. The components were dissolved in DMEM or MV at 37 °C in a water bath while gently shaking. Dulbecco’s modified eagle’s medium-based hydrogels were used by default for all cell populations. The hydrogels were centrifuged at 1000 rpm for 3 min to remove any bubbles. Subsequently, 8 × 10^6^ VEC dissolved in 1 mL of MV or 8 × 10^6^ VIC dissolved in 1 mL of DMEM were used to prepare VEC- or VIC-laden hydrogels with 2% (*w*/*v*) gelatin and 8% (*w*/*v*) gelatin with a final concentration of 2 × 10^6^ cells/mL. In multicellular tissue constructs, cell-laden VEC and VIC hydrogels were not mixed but used and printed separately instead to ensure the correct 3D-architecture.

The 3D-bioprinting was performed with an extrusion-based 3D-bioprinter (3D-Bioplotter^®^ Developer Series, EnvisionTec, Gladbeck, Germany) as previously described [12]. In summary, sterile cell-laden VEC and VIC hydrogels were transferred to individual syringes (7012134, Optimum^®^, Nordson EFD, Nordson Corporation, Westlake, OH, USA) with 25 G nozzles (7018391 Optimum^®^, Nordson EFD, Nordson Corporation, Westlake, OH, USA). The structures were printed on six-well cell culture plates in either a parallel line or honeycomb pattern with 25 G printer nozzles. For the parallel line pattern, a single continuous strand was printed which was arranged in parallel lines to fill the entire well, with the lines not touching each other, leaving open channels between them. On the contrary, the honeycomb pattern consists of strands in hexagonal combs that are strung together to fill the entire well. The printing patterns used are shown in the corresponding figures.

The platform temperature was maintained at T_P_ = 5 °C and the printing temperature T_H_ = 34 °C. The printing pressure between P = 400 hPa and P = 1000 hPa and the nozzle speed between v =10 mm/s and v = 20 mm/s were used. To mimic the 3D-architecture of the native aortic valve, VEC and multicellular 3D-architectures (VEC and VIC combined) were printed. The strands were cross-linked directly after the printing process with a 2% calcium chloride solution for 5 min and rinsed three times with DPBS. Tissue constructs were incubated in cell culture medium (DMEM, MV or a mixture) at 37 °C and 5% CO_2_ for up to 21 days. The culture medium was changed three times a week.

To evaluate the cell viability of 3D-printed tissue constructs, commercially available and established Cell Counting Kit-8 assay (CCK-8, 96992, Sigma-Aldrich, St. Louis, MO, USA) and live/dead staining with Calcein-AM and Ethidium-Homodimer-1 (L3224, Invitrogen™, ThermoFisher Scientific, Waltham, MA, USA) were used following the manufacturer’s protocols using a microplate reader (Infinite^®^ M1000 Pro, Tecan Group, Männedorf, Switzerland) and a laser scanning microscope (LSM 700, Carl Zeiss AG, Oberkochen, Germany), respectively. Quantification of live/dead stained images was performed with Fiji (ImageJ2, Version 2.9.0/1.53t) [34].

Statistical analysis was conducted using Prism 6 (GraphPad Software, La Jolla, CA, USA). For continuous variables, unpaired nonparametric Mann–Whitney and Kruskal–Wallis tests and Dunn’s multiple comparison tests were used, if applicable. All results are shown as mean values with a standard deviation and two important digits. The differences between the groups were defined as statistically significant for *p* < 0.05.

## 3. Results

### 3.1. Valvular Endothelial Cells Retain Good Long-Term Cell Viability after 3D-Bioprinting

**Figure 2:**
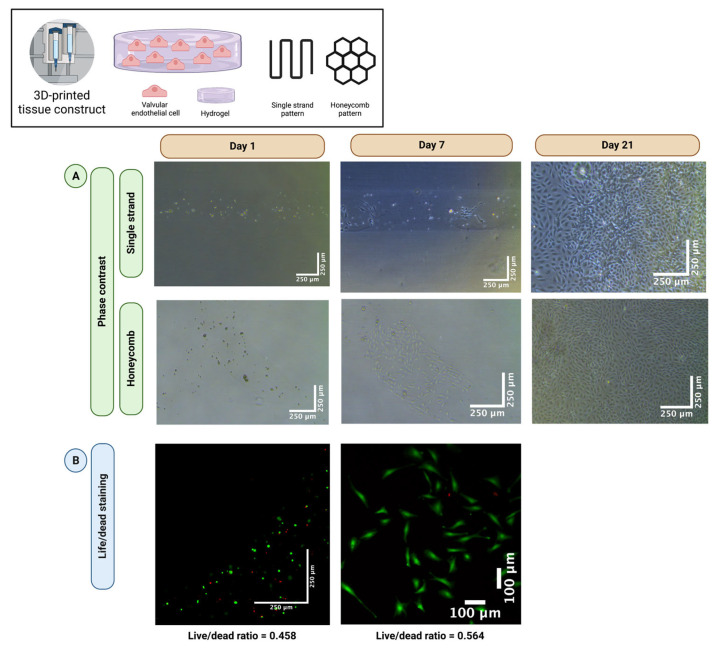
The 3D-bioprinting of valve endothelial cells. Ovine aortic valve endothelial cells (VEC) were isolated and single-layer cell-laden hydrogels consisting of 2% alginate and 8% gelatin in endothelial cell growth medium (MV) were printed with a 3D-bioprinter in a single strand or honeycomb pattern. The VEC-laden tissue constructs were incubated in MV for up to 21 days. Phase contrast images (**A**) revealed a resumption of cell division after seven days. After 21 days, all tissue constructs dissolved, and cells were confluent with the entire surface of the multi-well cell culture dish. A representative tissue construct is displayed for each group with phase contrast. Live/dead staining confirmed good cell viability (**B**). Calcein-AM (green dye) marks living and Ethidium-Homodimer-1 (red dye) apoptotic cells.

To evaluate whether VEC survive the cell-damaging process of 3D-bioprinting, single-layer VEC-laden hydrogels (2% alginate and 8% gelatin in MV) were printed in a single strand (*n* = 5) and honeycomb pattern (*n* = 5) and incubated in MV for up to 21 days. Figure 2 shows representative sections of phase contrast images (Figure 2A) and live/dead staining (Figure 2B) at different times. After seven days of incubation, the resumption of VEC cell division was observed regardless of the printed pattern. However, in the following course, all printed tissue constructs dissolved within the culture medium and the VEC migrated outside the former structure (Figure 2A, day 21), indicating disadvantages of the stability of the strand of MV-based hydrogels. Quantification of live/dead staining showed 45.8% living cells on day 1 and 56.4% living cells after 7 days. Therefore, in the following course, DMEM-based hydrogels were used for both VIC and VEC. However, these results confirm the general feasibility of 3D-bioprinting of vulnerable VEC with good long-term cell viability after printing.

### 3.2. The DEMEM/MV Ratio of 70:30 Is Best for Both Valvular Interstitial and Endothelial Cells

**Figure 3:**
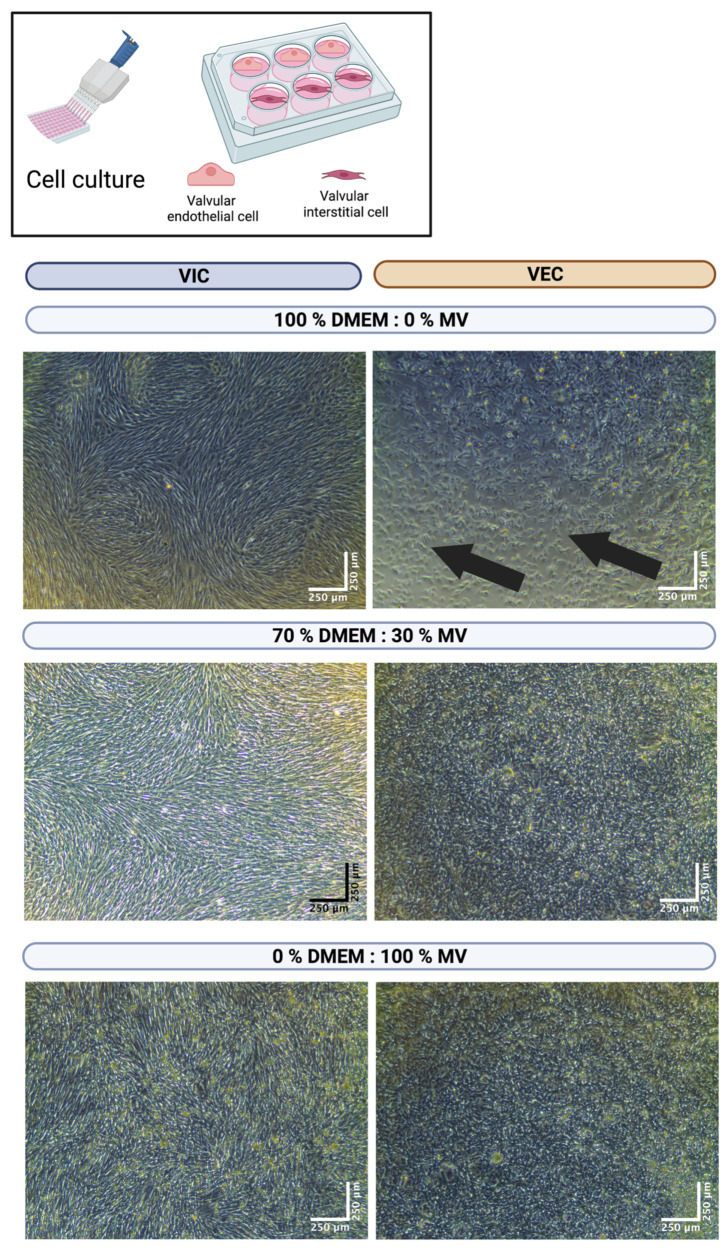
Cultivation of valve interstitial and endothelial cells with different media ratios. Ovine aortic valve interstitial (VIC, *n* = 11) and endothelial cells (VEC, *n* = 11) were isolated and seeded in individual multi-well culture dishes. Cells were incubated with different ratios of Dulbecco’s modified eagle’s medium (DMEM) and endothelial cell growth medium (MV). A representative image after seven days of incubation is displayed for different media concentrations with phase contrast. The arrows indicate areas of non-confluent cells.

As VIC and VEC have different nutritional demands, in general, different cell culture media (DMEM and MV, respectively) are used for cell culture. To evaluate the best media ratio for multicellular co-culture of both VIC and VEC, several media compositions were examined. First, VIC and VEC were seeded as monocultures in individual multi-well cell culture dishes (*n* = 11 wells each) and incubated for seven days in different media ratios, ranging from 100% DMEM and 0% MV to 0% DMEM and 100% MV in 10% steps (100/0%, 90/10%, 80/20%, etc.) (Figure 3). After seven days, the VICs were confluent and completely filled the wells, regardless of the concentration of DMEM in the medium. On the contrary, the VECs were only completely confluent after seven days with an MV ratio of at least 30%. No differences were observed between a medium composition of 70% DMEM and 30% MV up to 0% DMEM and 100% MV. As the dissolving of hydrogels was associated with the incubation with MV, the lowest possible MV concentration (namely 30% MV) in the cell culture medium was considered desirable.

### 3.3. The DEMEM/MV Ratio of 70:30 Is Suitable for 3D-Bioprinted Valvular Interstitial and Endothelial Cell Monoculture

**Figure 4:**
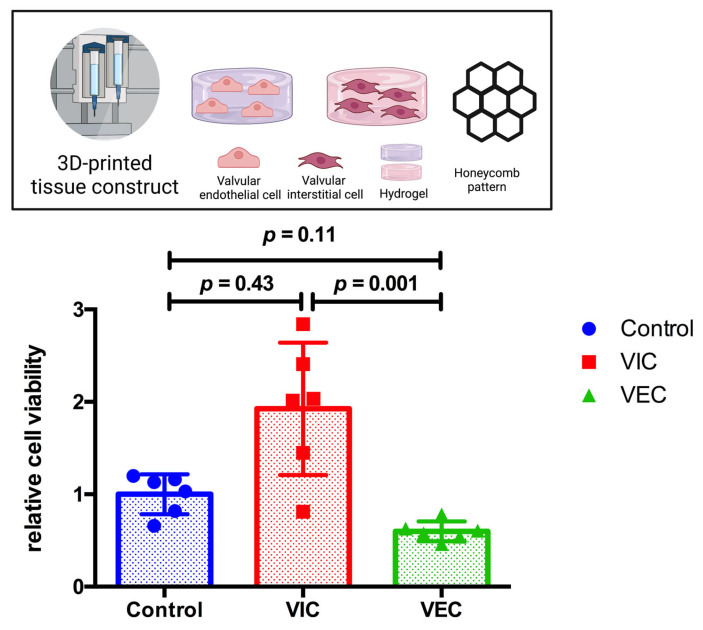
Relative cell viability of 3D-bioprinted VIC and VEC tissue constructs after 21 days. Ovine aortic valve interstitial cells (VIC) and endothelial cells (VEC) were printed with a 3D-bioprinter as a monoculture and incubated with 70% Dulbecco’s modified eagle’s medium (DMEM) and 30% endothelial cell growth medium (MV) for 21 days. Printed tissue constructs were compared to VIC tissue constructs incubated with 100% DMEM (control). Relative cell viability was examined using the cell counting kit-8 (CCK-8) assay on day 21 (*n* = 6 for each group). The measured values were standardized with the relative cell viability values of the control group. The results indicate the number of living cells in the tissue constructs that depend both on the number of cells damaged during the 3D-bioprinting process, as well as the composition of the cell culture medium examined and its effect on the resumption of regular cell division during post-printing incubation.

To prove the favorable results of a 70% DMEM and 30% MV medium composition and for 3D-bioprinted tissue constructs, both VIC- and VEC-laden hydrogels (2% alginate and 8% gelatin in DMEM) were printed in an individual three-layer honeycomb pattern and cultivated for 21 days. Cells were incubated in 70% DMEM and 30% MV and compared to VIC control tissue constructs in 100% DMEM (*n* = 6 for each group). The CCK-8 assay showed no difference in relative cell viability between the control tissue constructs and both the VIC tissue constructs (*p* = 0.43) and the VEC tissue constructs (*p* = 0.11) (Figure 4). However, the relative cell viability of 3D-bioprinted VIC-laden tissue constructs was about three times higher than VEC (*p* < 0.01).

### 3.4. The DEMEM/MV Ratio of 70:30 Is Suitable for 3D-Bioprinted Multicellular Valvular Interstitial and Endothelial Cell Co-Culture

**Figure 5:**
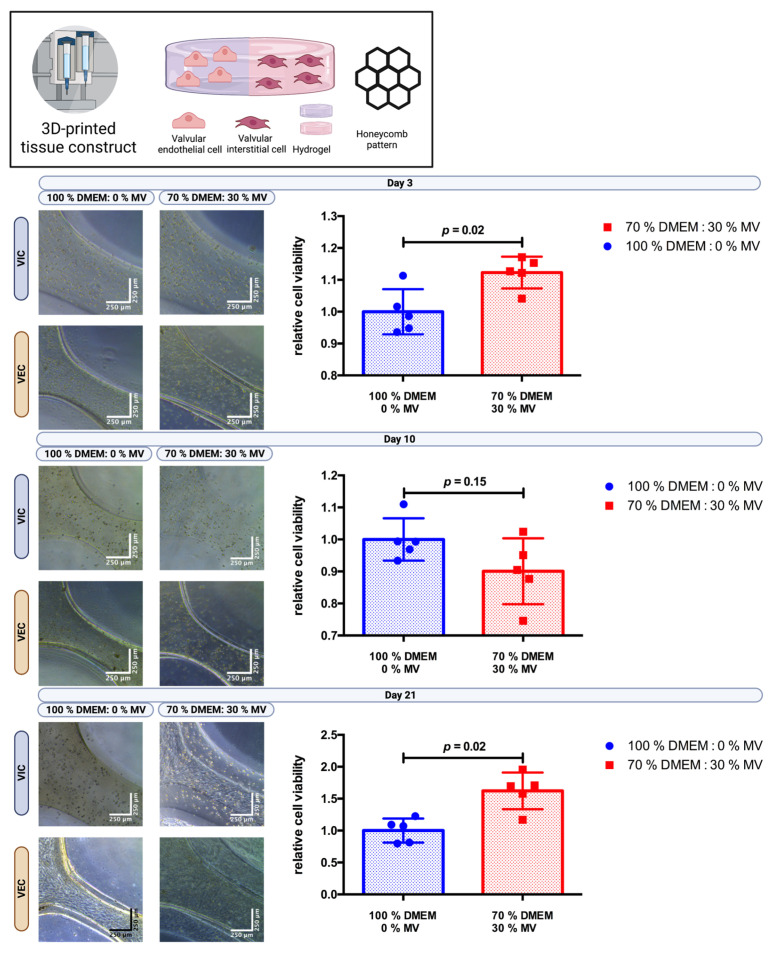
Cultivation of 3D-bioprinted multicellular tissue constructs of valve interstitial and endothelial cells with different media ratios. Ovine aortic valve interstitial cells (VIC) and endothelial cells (VEC) were printed with a 3D-bioprinter in a multicellular combined tissue constructs as a co-culture and incubated with different ratios of Dulbecco’s modified eagle’s medium (DMEM) and endothelial cell growth medium (MV). The printed tissue constructs were designed as a circle with a honeycomb pattern consisting of a semicircle of each VIC- and VEC-laden hydrogel. A representative image after three, ten, and twenty-one days of incubation is displayed for each group with phase contrast. Relative cell viability was examined using a cell counting kit-8 (CCK-8) assay on day three, ten, and twenty-one (*n* = 5 for each group). The measured values were standardized with the relative cell viability values obtained with 100% DMEM culture medium.

VIC- and VEC-laden hydrogels (2% alginate and 8% gelatin in DMEM) were processed to print combined multicellular tissue constructs to confirm the previous results. The tissue constructs were designed as a three-layer honeycomb pattern with half of the tissue construct consisting of a VIC-laden hydrogel and the other half of a VEC-laden hydrogel. A total of *n* = 30 multicellular tissue constructs were printed and incubated in 100% DMEM or 70% DMEM and 30% MV for up to 21 days. Cell viability was evaluated by the CCK-8 assay after three, ten, and twenty-one days (Figure 5). After 21 days of incubation, relative cell viability increased by approximately 60% (*p* = 0.02) for the 70:30 (DMEM/MV) medium ratio compared to just DMEM, indicating favorable results with respect to long-term post-printing cell viability of multicellular tissue constructs.

### 3.5. Multicellular Valvular Interstitial and Endothelial Cell-Laden Tissue Constructs Retain Cell Viability in Different 3D-Architectures

**Figure 6:**
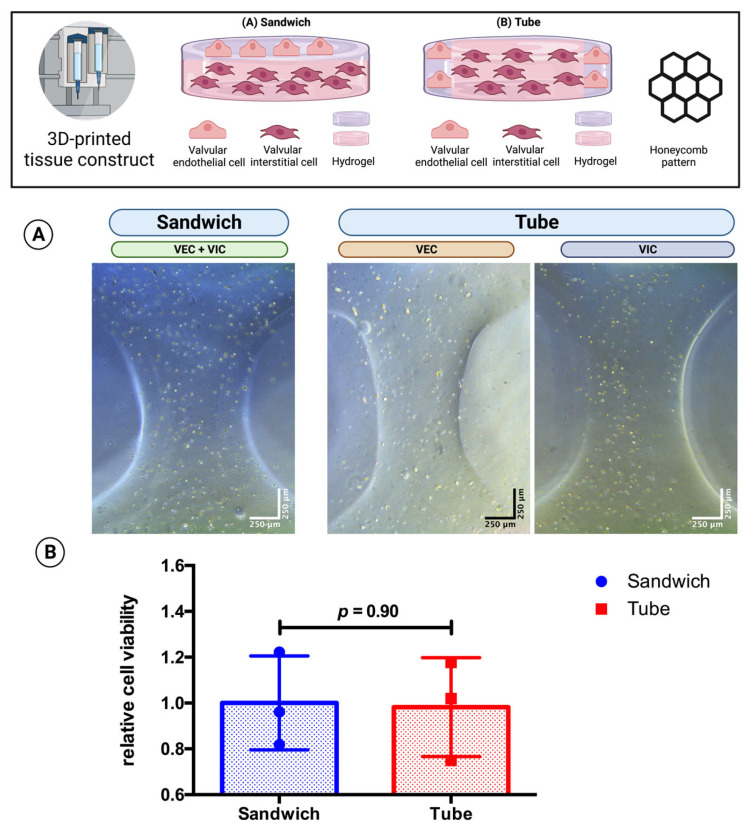
Comparison of 3D-architecture of 3D-bioprinted multicellular tissue constructs. Ovine aortic valve interstitial cells (VIC) and endothelial cells (VEC) were printed with a 3D bioprinter in a combined multicellular tissue constructs as a co-culture. Tissue constructs with two layers of VIC-laden hydrogel covered by a single layer of VEC-laden hydrogel (“sandwich” design) were compared with tissue constructs consisting of the center of the VIC-laden hydrogel and an outer boundary layer of the VEC-laden hydrogel (“tube”’ design) with the same volume and cell concentration. The multicellular tissue constructs were incubated with 70% Dulbecco’s modified eagle’s medium (DMEM) and 30% endothelial cell growth medium (MV) for 21 days. (**A**) representative image after one day of incubation displayed for each group with phase contrast. The image of the sandwich design shows an overlay of VIC and VEC; for the tube design, different parts of the tissue construct containing either VEC or VIC are displayed. (**B**) the relative cell viability was examined by a cell counting kit-8 (CCK-8) assay on day 21 (*n* = 3 for each group). The measured values were standardized to the relative cell viability values of the sandwich design.

Three-dimensional bioprinting enables the possibility of designing different 3D architectures of the cell-laden tissue constructs. As VEC cover the native aortic valve while VIC are found in the interstitial space within (Figure 1), different tissue constructs were designed to mimic this architecture. A “sandwich” design with VEC-laden hydrogel on top of two layers of VIC-laden hydrogel was compared with a “tube” where VEC-laden hydrogel was printed as an outer boundary layer around the VIC-laden hydrogel in the center of the tissue constructs (hydrogels consisting of 2% alginate and 8% gelatin in DMEM and printed in a honeycomb pattern with the same volume and cell concentration). The tissue constructs (*n* = 3 each) were incubated in 70% DMEM and 30% MV for 21 days. The CCK-8 assay showed similar relative cell viability (sandwich: relative cell viability = 1.00; tube: relative cell viability = 0.98; *p* = 0.90) for both 3D-architectures (Figure 6).

### 3.6. The Printing of Thick Multilayer Multicellular Valvular Interstitial and Endothelial Cell-Laden Tissue Construct Is Feasible

**Figure 7:**
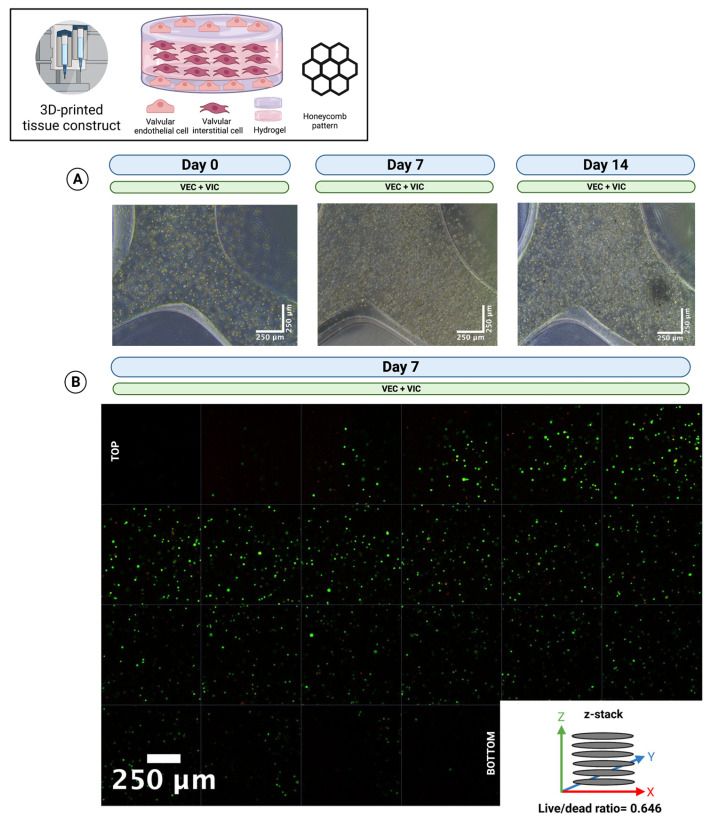
The 3D-bioprinting of thick multi-layer multi-cellular tissue constructs. Ovine aortic valve interstitial cells (VIC) and endothelial cells (VEC) were printed with a 3D-bioprinter in a combined multicellular tissue constructs as a coculture. Tissue constructs with three layers of VIC-laden hydrogel covered by a single layer of VEC-laden hydrogel on each side were incubated with 70% Dulbecco’s modified eagle’s medium (DMEM) and 30% endothelial cell growth medium (MV) for up to 14 days. (**A**) Representative image directly after printing (day 0), after 7 and 14 days of incubation, is displayed with phase contrast. The images show an overlay of VIC and VEC. (**B**) Live/dead staining after 7 days of incubation. The image shows a z-stack of a representative section from the top to the bottom of the multilayer tissue construct. Calcein-AM (green dye) marks living and Ethidium-Homodimer-1 (red dye) apoptotic cells.

To prove long-term cell viability for thick multilayer multicellular 3D-printed tissue constructs, constructs were printed in a modified “sandwich” design consisting of three layers of VIC-laden hydrogel covered by a single layer of VEC-laden hydrogel on both sides in a honeycomb pattern (*n* = 5) and incubated for up to 14 days in 70% DMEM and 30% MV. Phase contrast microscopy (Figure 7A) confirmed stable tissue constructs throughout the whole incubation period. In addition, live/dead staining (Figure 7B) revealed viable cells throughout all layers of the entire thick multicellular tissue construct (64.6% living cells).

## 4. Discussion

Aortic valve pathologies are responsible for a substantial global burden of disease. In particular, CAVD, with its complex and still not fully understood pathogenesis, requires an intensive and continuous research effort. Novel 3D in vitro models may help further examine the complex interaction of VIC, VEC, and ECM in the progression of CAVD. In this research project, as a proof of principle, we set ourselves the goal of expanding our previously reported 3D-printed model to include the cell population of the VEC and to bring this model closer to the native valve. We were able not only to show that 3D-bioprinting of vulnerable VEC is feasible but the combined bioprinting of multicellular tissue constructs with distinct 3D-architectures of both VIC and VEC with preserved long-term cell viability can be achieved successfully. In this context, combining DMEM-based cell-laden VIC and VEC hydrogels processed with low to medium pressure printing techniques and incubation with a medium composition of 70% DMEM and 30% MV could overcome both problems of strand precision and durability, as well as cell viability within multilayer multicellular tissue constructs.

By now, 3D-bioprinted in vitro models of the aortic valve have omitted VEC and focused instead on VIC, as they are generally easier to handle and cultivate [9,10,11,12]. However, several studies have reported 3D-bioprinted tissue constructs that use endothelial cells but not aortic valvular endothelial cells [18,19,20,35]. Instead, these studies have employed stem-cell-derived endothelial cells or HUVEC, which is different from our approach with adult cells isolated from native valves. However, endothelial cells were dissolved in hydrogel and printed as cell-laden tissue constructs and then incubated in cell culture medium as our approach [18,19,35]. Most importantly, endothelial cells overcame the harmful printing process and resumed cell division and proliferation in the following course.

In our current model, we used a co-culture set-up involving VIC and VEC to develop a multicellular tissue construct allowing the cells to communicate with each other. In line with that, Duan et al. previously also reported a 3D-bioprinted tissue construct with a co-culture of two different cell populations for studying aortic valve biology [9]. However, they used VIC and smooth muscle cells derived from aortic root sinus and not VEC [9]. Furthermore, the tissue constructs were only grown for up to seven days [9]. Other groups have combined HUVEC and aortic smooth muscle cells or seeded cardiomyocytes on 3D-bioprinted HUVEC constructs [20,35]. Although the latter works represent multicellular tissue constructs, in our opinion, they cannot yet mimic the aortic valve [20,35].

However, several studies have previously reported successful co-culture of both VEC and VIC for in vitro CAVD research [26,27,28,29,30,31,32,33]. Although most co-culture models used 3D-cell culture, 3D-bioprinting has not yet been established in this context [28,29,30,31,32,33]. However, 3D-bioprinting offers several advantages compared to conventional 3D-cell culture, including fast, precise, and reproducible scaffold production by an automated process [36]. We have identified a medium composition of 70% DMEM and 30% MV to be ideal as it significantly increased the long-term cell viability of our multicellular tissue constructs by meeting the different nutritional requirements of VIC and VEC. In particular, while VIC showed good proliferation and cell division even incubated in 100% MV, the proliferation and cell division of VEC was dependent on incubation with at least 30% MV. However, MV was associated with dissolved strands; therefore, we used DMEM hydrogels and the lowest possible MV concentration for our experiments. Interestingly, three of the mentioned previous studies used only DMEM for the incubation of the multicellular tissue constructs; another three studies employed a medium composition of 50% DMEM and 50% endothelial cell medium, and two studies used only endothelial cell medium [28,29,30,31,32,33]. However, none of them explained the rationale behind the medium used for the co-culture of VIC and VEC. Cells were cultured for 3 to 28 days, and most studies incubated the co-culture for 7 to 14 days to examine the cell–cell interaction and the endothelial-to-mesenchymal transition [28,29,30,31,32,33]. On the contrary, in the present work, we focused on the methodical aspects, feasibility, and achievement of long-term cell viability of 3D-bioprinted multicellular tissue constructs combining VIC and VEC for CAVD. In general, adequately addressing nutritional demands is crucial to achieve long-term cell viability, especially in thick multilayer cellular constructs [12,37]. In particular, partial hydrogel degradation is needed in order to create enough space for extracellular matrix production, cell division, and migration in the cellular construct [37]. Although we did not experience problems with cell nutrition in the current project, to overcome potential problems of cell aggregates suffering from nutritional gradients in 3D-cell culture, bioprinting of perfusable constructs may offer a variety of new perspectives [37,38,39,40,41]. Designing complex perfusable structures in the scaffold may also allow us to perfuse different parts of multicellular constructs with different culture medium to enhance the cellular nutrition compared to the usage of a medium mixture as in the current project.

As the described model of a 3D-bioprinted multicellular aortic valve tissue construct has not been achieved before, we particularly focused on the technical proof of principle of the constructs and long-term post-printing cell viability instead of the molecular biological characterization of the cells. Although we were able to demonstrate viable cells in the 3D-bioprinted tissue constructs, we did not examine the functional status of the cells that limits the validity of our data. However, as we printed VIC and VEC in both single and multicellular tissue constructs as well with defined spatial resolution, we were able to test the post-printing and long-term viability for both cell lines individually [12]. A detailed examination of the key markers and functional status of cells is planned for future research projects. In this context the endothelial-to-mesenchymal transition that plays an important role in the pathogenesis of cardiovascular diseases is of particular interest [42]. This phenomenon describes the transition of endothelial cells such as VEC to a mesenchymal-like phenotype, e.g., myofibroblast-like [42,43,44]. These cells can also differentiate into osteo- or chondrocyte-like cells and are associated with CAVD [42,43,44]. Transforming growth factor beta (TGF-β) has been shown to be an important mediator of the endothelial-to-mesenchymal transition and its signaling cascade can be activated by endothelial shear stress [42,45]. As 3D-bioprinting also induces shear stress, an unintentional concomitant induction of endothelial-to-mesenchymal transition cannot be ignored and should be the topic of future research applications in the context of 3D-bioprinting and CAVD [15,16,17].

In the current project, we used only the hydrogel composition of gelatin and alginate. These hydrogels are commonly used for 3D-bioprinting as they are relatively cheap and easy to process [46]. By now, a large variety of different hydrogels have been described in the literature [46]. These hydrogels all differ in terms of their respective printing properties, biocompatibility, and cost effectiveness [46]. We used gelatin and alginate dissolved in DMEM for multicellular tissue constructs as we were able to report favorable results for the 3D-bioprinting of VIC before [12]. However, compared to hydrogels with physiological components of the ECM, such as collagen hydrogels, the processed gelatin/alginate hydrogel probably falls short in terms of biocompatibility [47]. In addition to collagen hydrogels, another interesting and promising option may be the use of silk fibroin for 3D-bioprinting [48].

We dissolved both VEC and VIC in individual hydrogels and printed them with two different printing heads in an automatic process by the 3D-bioprinter. In contrast to that, attaching the VEC to the hydrogel after the printing process seems to be another possible technical aspect. This might help align the VEC directly on the outside boundary of the hydrogel. However, in our opinion, this technique is likely less reproducible, leading to differences between the different manufactured multicellular tissue constructs. In contrast to that, with the reported technique, we were able to use an automated single-step method, which should be faster and easier to handle.

Future projects may now focus on mimicking the native valve ECM as the next missing piece among the structural components of the aortic valve. Hereby, more physiological and biocompatible hydrogels reproducing the native ECM should amend the reported model. The ECM is an elementary component in the pathogenesis of CAVD as an inflammatory reaction in the ECM promotes remodeling and changes in collagen architecture with consecutive fibrosis and subsequent calcification [49,50,51,52]. To evaluate these processes for CAVD research, an even more advanced model would be beneficial. In addition, detailed analyses including gene expression, protein transcription, the occurrence of endothelial-to-mesenchymal transition of VEC, and the induction of calcification of the tissue constructs should be performed in the nearby future. The 3D-bioprinted multicellular tissue constructs may help broaden our existing knowledge of CAVD pathogeneses and potential treatment targets.

## 5. Conclusions

Our project is a proof of principle that 3D-bioprinting of multicellular tissue constructs composed of VECs and VICs is a novel, feasible approach to designing tissue constructs that mimic the structure of the native aortic valve to study aortic valve pathologies such as CAVD. By combining low- to medium-pressure printing methods, gelatin, and alginate hydrogels, as well as a composition of 70% DMEM and 30% MV mediums, both aortic valvular cell types proliferated at 21 days of incubation and maintained their long-term viability in different complex 3D-architectures. We successfully described a fast, cheap, and automated single-step approach to produce highly reproducible 3D-multicellular constructs of the aortic valve that allow us to observe and analyze mechanisms in the pathogenesis of CAVD for a sufficient period of time. Although several further improvements, including bioprinting hydrogels with ECM components, are needed for the perfect tissue model of the aortic valve, our reported model represents an important finding as it is the first reported 3D-bioprinted model of the aortic valve combining both VIC and VEC in a single multicellular construct.

## Data Availability

The datasets used and analyzed during the current study are available from the corresponding author on reasonable request.

## Abbreviations

CAVD	Calcific aortic valve disease
DMEM	Dulbecco’s modified eagle’s medium
DPBS	Dulbecco’s balanced salt solution
ECM	Extracellular matrix
HUVEC	Human umbilical vein endothelial cell
MV	Endothelial cell growth medium
TGF-β	Transforming growth factor beta
VEC	Valvular endothelial cell
VIC	Valvular interstitial cell
